# Critical Assessment of Single-Use Ureteroscopes in an *In Vivo* Porcine Model

**DOI:** 10.1155/2020/3842680

**Published:** 2020-04-27

**Authors:** Brian Ceballos, Charles U. Nottingham, Seth K. Bechis, Roger L. Sur, Brian R. Matlaga, Amy E. Krambeck

**Affiliations:** ^1^Indiana University School of Medicine, Department of Urology, 1801 Senate Blvd. Suite 220, Indianapolis, IN 46202, USA; ^2^University of California San Diego School of Medicine, Department of Urology, 200 West Arbor Drive #8897, San Diego, CA 92103, USA; ^3^Johns Hopkins University School of Medicine, James Buchanan Brady Urological Institute, 600 North Wolfe Street Park 2 Room 221, Baltimore, MD 21287, USA

## Abstract

**Methods:**

A female pig was placed under general anesthesia and positioned supine, and retrograde access to the renal collecting system was obtained. The LithoVue (Boston Scientific) and Uscope (Pusen Medical) were evaluated by three experienced surgeons, and each surgeon started with a new scope. The following parameters were compared between each ureteroscope: time for navigation to upper and lower pole calyces with and without implements (1.9 F basket, 200 *μ*m laser fiber, and 365 *μ*m laser fiber for upper only) in the working channel and subjective evaluations of maneuverability, irrigant flow through the scope, lever force, ergonomics, and scope optics.

**Results:**

Navigation to the lower pole calyx was significantly faster with LithoVue compared to Uscope when the working channel was empty (24.3 vs. 49.4 seconds, *p* < 0.01) and with a 200 *μ*m fiber (63.6 vs. 94.4 seconds, *p*=0.04), but not with the 1.9 F basket. Navigation to the upper pole calyx was similar for all categories except faster with LithoVue containing the 365 *μ*m fiber (67.1 vs. 99.7 seconds, *p*=0.02). Subjective assessments of scope maneuverability to upper and lower pole calyces when the scope was empty and with implements favored LithoVue in all categories, as did assessments of irrigant flow, illumination, image quality, and field of view. Both scopes had similar scores of lever force and ergonomics.

**Conclusions:**

In an *in vivo* porcine model, the type of single-use ureteroscope employed affected the navigation times and subjective assessments of maneuverability and visualization. In all cases, LithoVue provided either equivalent or superior metrics than Uscope. Further clinical studies are necessary to determine the implications of these findings.

## 1. Introduction

Renal stone disease is a growing concern for patients in the United States, which makes optimal management increasingly important. Between 1994 and 2007, there was an increase in incidence in renal stones according to the National Health and Nutrition Examination Survey (NHANES) [1]. The increase was estimated to be from 6.3% to 10.6% in men and from 4.1% to 7.1% in women [2]. Despite the various methods of management for uncomplicated urolithiasis, ureteroscopy has become widely accepted as a desirable form of treatment owing to its efficacy, efficiency, ability to be performed in an outpatient setting, and minimal side effect profile [3].

A more recent advancement in the surgical treatment of urolithiasis has been the use of disposable (non-reusable) ureteroscopes as opposed to the traditional reusable ureteroscopes [4]. In October 2015, Boston Scientific introduced LithoVue, the first disposable flexible ureteroscope in efforts to avoid maintenance costs and loss of deflection with repeated use, as this was a central concern for the reusable ureteroscopes [5].

Looking forward, the difference in safety and efficacy between single-use and reusable ureteroscopy becomes essential to understand when considering the management of stone disease and cost of treatment. It is estimated that urolithiasis will cost the healthcare economy up to $3 billion by the year 2030 [6], and it is unclear exactly how integration of disposable ureteroscopes will impact costs going forward. To date, the studies between disposable ureteroscopes are limited, as only a handful of scopes are available for clinical use. The objective of this study is to compare maneuverability, navigation time, and visualization between two disposable ureteroscopes in a porcine model: the Boston Scientific LithoVue and Pusen Uscope.

## 2. Methods

The study was conducted at CBSET, Inc. (Lexington, MA), under IACUC protocol #I00240. A female pig weighing approximately 35 kg was placed under general anesthesia and positioned supine, and retrograde access to the renal collecting system was obtained cystoscopically. An 11/13 ureteral access sheath was placed under fluoroscopic guidance, with proximal positioning in the proximal ureter. Ureteroscopy was performed by three experienced endourologists (SB, RS, and AK) with both the LithoVue (Boston Scientific, Boston, MA) and the first-generation Uscope (Zhuhai Pusen Medical Co., Zhuhai, Guangdong, China). Each ureteroscope had a tip diameter of 9.5 French as well as a working channel that measured 3.6 French in size. Each surgeon started with a new scope, but they were not formally blinded due to the fact that they each had prior experience with the LithoVue ureteroscope. For all individual scopes, each surgeon completed seven total tasks five times each, for a total of 35 passes of a given scope.

The time required for navigation to upper and lower pole calyces was recorded in seconds. These tasks were assessed with and without implements placed through the working channel. The implements used were a 200 *μ*m holmium:YAG laser fiber (Flexiva, Boston Scientific) and a 1.9 French nitinol basket (Zero Tip, Boston Scientific). An additional implement of a 365 *μ*m holmium:YAG laser fiber (Flexiva, Boston Scientific) was also used, but the scopes were only passed to the upper pole with this fiber. Following completion of these tasks, each surgeon gave a subjective assessment of scope maneuverability on a 0–5 scale, with 0 being the lowest maneuverability score and 5 being the highest.

Upon completion of all 35 passes for a given scope, each surgeon then gave a subjective assessment of irrigant flow through the scope, lever force, ergonomics, and scope visualization at the completion of all passes of each scope, thereby representing visualization at only the very end of the scope's life span. The visualization included three categories: image quality, illumination, and field of view. These assessments were also graded on a 0–5 scale, with 0 being the lowest visualization score and 5 being the highest.

Mean times and scores were calculated and compared between the LithoVue and Uscope for each parameter using two-sided student's *t*-test. Statistical analysis was performed using SPSS version 25.0 (Armonk, NY).

## 3. Results

Each pass of the ureteroscope by each surgeon for all parameters was included for analysis. Each surgeon successfully performed a total of 35 passes with each ureteroscope.

The results of navigation times to upper and lower pole calyces are summarized in Table 1. Navigation to the lower pole calyx was significantly faster with LithoVue compared to Uscope when the working channel was empty (24.3 vs. 49.4 seconds, *p* < 0.01) and with a 200 *μ*m fiber (63.6 vs. 94.4 seconds, *p*=0.04), but not with the 1.9 F basket (58.2 vs. 70.1 seconds, *p*=0.18). Navigation to the upper pole calyx was not significantly faster in either scope with the working channel was empty, with a 1.9 F basket, and with a 200 *μ*m fiber. However, upper pole navigation was faster using LithoVue with a 365 *μ*m fiber (67.1 vs. 99.7 seconds, *p*=0.02).

Subjective maneuverability assessments of each ureteroscope are summarized in Table 2. LithoVue had significantly higher assessment scores than Uscope for navigation to both upper and lower poles when the working channel was empty and when it contained all three implements.

The subjective assessment of irrigant flow, lever force, ergonomics, and scope optics upon completion of scope usage is presented in Table 3. Both scopes had statistically similar scores for lever force and ergonomics. LithoVue had significantly higher ratings than Uscope in the categories of irrigant flow (5.0 vs. 3.3, *p*=0.01), illumination (4.0 vs. 1.3, respectively; *p*=0.02), image quality (4.0 vs. 1.3, respectively; *p*=0.02), and field of view (4.7 vs. 1.3, respectively; *p* < 0.01).

**Figure 1:**
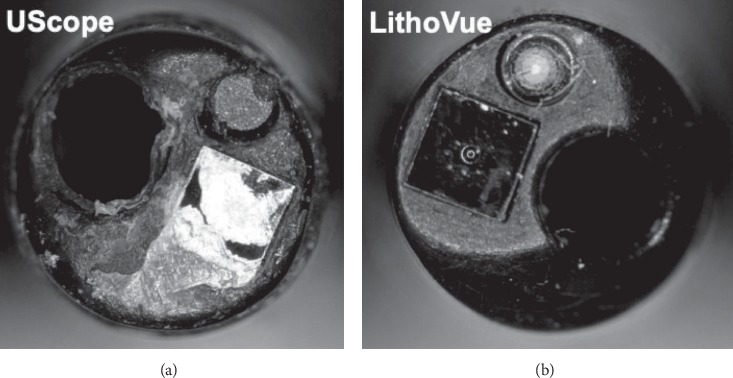
Representative photographs of the tip of the Uscope (a) and LithoVue (b) following completion of all tasks (total of 36 passes per scope).

Figure 1 shows representative photographs of the tip of each ureteroscope following completion of all 35 passes.

## 4. Discussion

The purpose of this study was to compare differences between the Boston Scientific LithoVue and Pusen Uscope, two commonly used disposable ureteroscopes in a porcine model. After 35 passes with each type of ureteroscope by all three surgeons, it was determined that the LithoVue provided either equivalent or faster navigation time to the renal calyces, and that the subjective visualization was superior when compared to the Uscope. To our knowledge, this is the first critical comparison between these two ureteroscopes in a porcine model.

In comparison to the current literature, our results regarding the disposable ureteroscopes appear similar in nature. Although we did not evaluate resolution at specified distances, Winship and colleagues reported superior resolution using the LithoVue in comparison to the Uscope when evaluated at 10 mm [7]. When the LithoVue was compared to the Uscope and the reusable Flex-X2 ureteroscope by Marchini et al, they found that the LithoVue was superior to both the Uscope and Flex-X2 in regards to optical resolution, field of view, deflection capacity, and irrigation when working with larger instruments in the working channel [8]. These studies support the potential benefits of the disposable ureteroscopes, as optimal resolution plays an essential role in safety during ureteroscopy.

While costs often vary between regions and institutions, the price of ureteroscopy is an important factor that should be taken into account when comparing ureteroscopes. According to Salvadó et al., the disposable ureteroscopes are 32 times cheaper than the overall costs of a reusable ureteroscope in Chile [9]. Between disposable ureteroscopes, the Uscope is about 42% cheaper than the LithoVue [9]. However, the LithoVue is the only disposable ureteroscope that has a one-ureteroscope-per case guarantee, which ensures that Boston Scientific will replace the ureteroscope at no additional cost to the patient or care facility if a LithoVue ureteroscope breaks during a procedure [7].

Another factor to consider when comparing ureteroscopes is rates of malfunction. Because each disposable ureteroscope typically costs >$800 per case, rates of failure become an integral part in cost analysis [4]. In our study, we had no malfunction of either disposable ureteroscope that required replacement during a case, which is consistent with the experience of Salvadó et al. who did not experience malfunction of the Pusen Uscope in their series of 71 cases [9]. However, in regards to the LithoVue, our results appear to be slightly improved to that of Usawachintachit et al., who experienced a rate of malfunction of 4.4% among their 129 cases [10]. However, it is once again worth noting that the one-ureteroscope-per-case policy previously outlined is only supported by Boston Scientific [7].

While there are limitations to this study, our aim is to provide insight towards our experience with the LithoVue and Uscope disposable ureteroscopes. One limitation is the difficulty with blinding for the type of ureteroscope used by the urologist. The design, color, and interface of each scope may be enough to influence the user's analysis. Another possible limitation is the inability to control for variable experience with each scope. While one surgeon may have ease with one ureteroscope, their lack of experience with the other may influence their functional use and analysis of the product. The value in this study is in its *in vivo* nature as it takes on a more practical approach to the analysis as opposed to benchtop analysis. Another strength of our study is the analysis by three surgeons of different institutions, as this helps reduce the bias that could result from similar practices within the same care facility.

## 5. Conclusion

Using an *in vivo* porcine model, the type of disposable ureteroscope significantly affected the navigation times and subjective visualization among three experienced urologists. Overall, the LithoVue proved to be either equivalent or superior to the Pusen Uscope.

## Figures and Tables

**Table 1:** Surgeon times (seconds) for navigation to upper and lower poles between LithoVue and Uscope when the working channel is empty, with a 1.9-French basket, a 200 *μ*m laser fiber, and a 365 *μ*m laser fiber (upper only).

Surgeon time in seconds	Empty working channel	1.9-French basket	200 *μ*m laser fiber	365 *μ*m laser fiber
Navigation to upper pole				
LithoVue	50.4 (31.5)	75.3 (32.7)	76.0 (37.6)	67.1 (23.7)
Uscope	35.7 (25.4)	106.8 (46.6)	90.1 (41.5)	99.7 (42.0)
*p* value	0.20	0.05	0.37	0.02

Navigation to lower pole				
LithoVue	24.3 (11.9)	58.2 (18.5)	63.6 (25.9)	—
Uscope	49.4 (31.4)	70.1 (27.4)	94.4 (47.8)	—
*p* value	0.01	0.18	0.04	—

The values are expressed as mean (SD).

**Table 2:** Subjective surgeon assessment of maneuverability between LithoVue and Uscope for navigation to upper and lower poles when the working channel is empty, with a 1.9-French basket, a 200 *μ*m laser fiber, and a 365 *μ*m laser fiber (upper only).

Subjective scope maneuverability assessment (0–5 rating, 5 = highest)	Empty working channel	1.9-French basket	200 *μ*m laser fiber	365 *μ*m laser fiber
Navigation to upper pole				
LithoVue	4.40 (0.51)	4.47 (0.52)	5.00 (0.00)	4.87 (0.35)
Uscope	3.80 (0.41)	3.47 (0.74)	3.00 (0.65)	2.23 (0.93)
*p* value	<0.01	<0.01	<0.01	<0.01

Navigation to lower pole				
LithoVue	4.33 (0.49)	4.67 (0.49)	5.00 (0.00)	
Uscope	3.40 (0.63)	3.60 (0.51)	3.00 (0.76)	—
*p* value	<0.01	<0.01	<0.01	—

The values are expressed as mean (SD).

**Table 3:** Subjective surgeon assessments of irrigant flow, lever force, ergonomics, and scope optics between LithoVue and Uscope after completion of 35 passes of the ureteroscope.

Subjective assessments (0–5 rating, 5 = highest)	LithoVue	Uscope	*p* value
Irrigant flow	5.0 (0.0)	3.3 (0.6)	0.01
Lever force	4.0 (1.0)	3.0 (1.7)	0.44
Ergonomics	4.7 (0.6)	3.0 (1.7)	0.19
Illumination	4.0 (1.0)	1.3 (0.6)	0.02
Image quality	4.0 (1.0)	1.3 (0.6)	0.02
Field of view	4.7 (0.6)	1.3 (0.6)	<0.01

The values are expressed as mean (SD).

## Data Availability

The data used to support the findings of this study are available from the corresponding author upon request.
